# The Optimization of Mix Proportion Design for SCC: Experimental Study and Grey Relational Analysis

**DOI:** 10.3390/ma15041305

**Published:** 2022-02-10

**Authors:** Xinxin Ding, Mingshuang Zhao, Xue Qiu, Yupu Wang, Yijie Ru

**Affiliations:** 1 International Joint Research Lab for Eco-Building Materials and Engineering of Henan, School of Civil Engineering and Communications, North China University of Water Resources and Electric Power, Zhengzhou 450045, China; zhaomingshuang@ncwu.edu.cn (M.Z.); xueqiu@stu.ncwu.edu.cn (X.Q.); yupuwang@stu.ncwu.edu.cn (Y.W.); yijieru@stu.ncwu.edu.cn (Y.R.); 2 Collaborative Innovation Center for Efficient Utilization of Water Resources, North China University of Water Resources and Electric Power, Zhengzhou 450045, China

**Keywords:** self-compacting concrete (SCC), mix proportion design, optimization, experimental verification, the grey relational analysis

## Abstract

The optimization of mix proportions based on the targeted fresh and hardened performances of self-compacting concrete (SCC) is a foundation for its transition from laboratory research to industrial production. In this paper, the mix proportions of various SCC mixtures were designed by the absolute volume method with changes in the content of river sand and manufactured sand, the content of fly ash and granulated ground blast furnace slag (GGBS) and the maximum particle sizes of coarse aggregates. This experimental study was carried out to verify the workability, density and cubic compressive strength of SCC. The results show that SCC demonstrated good performance with appropriate mix proportions of manufactured sand and river sand. A hybrid effect of fly ash and GGBS appeared on the fresh performance of SCC with a constant strength, and the coarse aggregate with a smaller maximum particle size was beneficial to the workability but detrimental to the compressive strength of SCC. Finally, the optimization of the mix proportion of SCC was evaluated by grey relational analysis, in which the weight of the indicators was determined by the entropy method to improve the evaluation credibility. As a result, the optimal mix proportions of SCC were selected.

## 1. Introduction

Self-compacting concrete (SCC) is a kind of high fresh-performance concrete that fills and compacts in a given framework by its own weight without other assistance [1]. As opposed to other conventional vibrated concretes, the defining feature of SCC is its superior workability. Besides the typical requirements of setting time, cohesiveness and water retention, fresh SCC should also satisfy stricter requirements of filling ability, passing ability and segregation resistance. Furthermore, there is a closed relationship between the workability of fresh SCC and the basic mechanical properties of hardened SCC [2,3]. The correct proportioning of self-compacting concrete mixes based on the target fresh and hardened performances is a hot research point.

The mix proportion requires the segregation and settlement of fresh SCC, a large number of binder materials, a higher sand ratio and a smaller maximum particle size of coarse aggregate in order to maintain a sufficient yield value, ensure the viscosity of the fresh mixes and, ultimately, to reduce bleeding [4,5,6,7]. A traditional method to ensure the equal performance of SCC is to add mineral materials. The presence of fly ash in SCC reduces its water demand and improves its desired workability but also decreases its compressive strength [7,8,9]. The presence of granulated ground blast furnace slag (GGBS) in SCC improves its fresh properties as well as its continuing strength development [10,11]. A positive effect can be observed in SCC with both GGBS and fly ash added in an appropriate proportion and dosage [12]. The passing ability and the segregation resistance of fresh SCC reduce with increasing coarse aggregate size from 9.5 mm to 19 mm, while the compressive strength of hardened SCC increases [13]. A greater amount of binder material content is required to obtain self-compacting workability for an SCC mixed with manufactured sand compared with an SCC mixed with river sand. This is due to the special morphology features of manufactured sand, such as its rough surfaces, irregular shapes, angular edges and inevitable stone powder [14,15,16]. The workability of fresh SCC could change with hybrid sand mixed with different contents of river sand and manufactured sand due to the changes of particle grading of the hybrid sand [17].

Numerous methods have been used to study the properties of concrete, such as laboratory tests [2,18], field monitoring [19], mathematical statistics [20], numerical analysis [21] and software simulation [22]. As the workability and strength of SCC are affected by multiple factors, it is unclear which one is the major factor. Therefore, the optimal mix proportion of SCC is difficult to obtain from original tests. This encourages research that takes concrete as a system with random, gray and fuzzy uncertain messages and tries to predict and evaluate its properties by grey relational analysis [23,24,25]. The grey relational analysis theory was founded in 1982 by Deng in China; it is an uncertain systematic theory dealing with a “small sample” and “poor information” [26]. As a type of system analytical technology, the basic principle of grey relational analysis is to evaluate the relationships of connections between sequences according to the geometric shape of a sequence curve. The closer the geometric shapes, the greater the correlation between the corresponding sequences and vice versa.

Based on the above statement, the optimization of the mix proportion of SCC is important to ensure its transition from laboratory research to industrial production. In this paper, an experimental verification was conducted for the mix proportion design of SCC with multiple factors, including the type and content of mineral admixtures, the type and content of sand and the maximum particle size of the coarse aggregate. The test results were evaluated by grey relational analysis to clarify the main influencing factors for both the workability and strength of SCC.

## 2. Mix Proportion Design of SCC

### 2.1. Raw Materials

**Figure 1:**
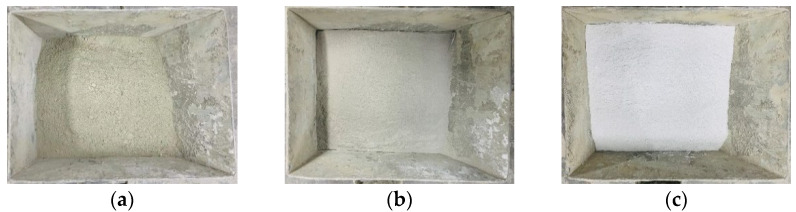
Pictures of binder materials: (**a**) cement; (**b**) fly ash; (**c**) GGBS.

Ordinary Portland cement, with a strength grade of 52.5, fly ash of class II, and ground granulated blast furnace slag (GGBS) of class S95, was used as a binder material. The detailed physical and mechanical properties of this cement are listed in Table 1, where it can be seen that it met the specification of China code GB 175 [27]. The detailed physical and mechanical properties of the fly ash and GGBS are listed in Table 2, where it can be seen that they met the specification of China code GB/T 51003 [28]. Pictures of the binder materials are presented in Figure 1.

**Figure 2:**
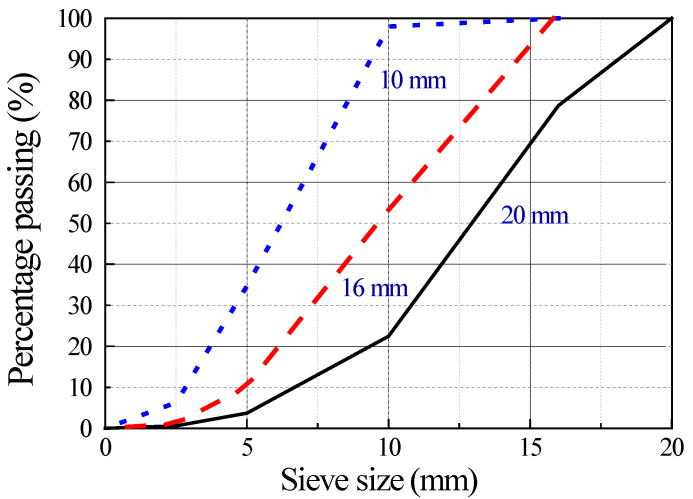
Particle size distribution of the coarse aggregate used.

**Figure 3:**
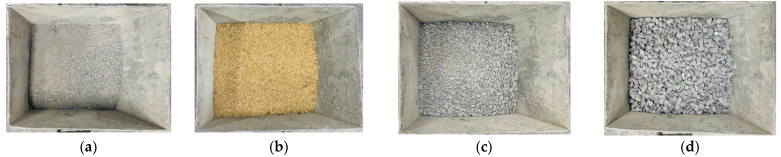
Pictures of aggregates: (**a**) manufactured sand; (**b**) river sand; (**c**) 5~10 mm aggregate; (**d**) 10~16 mm aggregate.

Manufactured sand crushed from limestone and river sand were used as fine aggregates. Their physical and mechanical properties are listed in Table 3. Crushed limestones with maximum particle sizes of 20 mm, 16 mm, and 10 mm from 1 company were used as coarse aggregates. Their physical and mechanical properties are listed in Table 4. Their particle gradations are presented in Figure 2 and are adjusted according to the principle of maximum close-packing density. The gradations of fine and coarse aggregates met the specifications of China codes GB/T 14684 and GB/T 14685 [29,30]. Pictures of the fine and coarse aggregates are presented in Figure 3.

A polycarboxylate-based superplasticizer was used as a water reducer with a water-reducing rate of 29% and solid content of 23%. The mixing water was tap water.

### 2.2. Mix Proportion Design

In this study, the absolute volume method is used to design the mix proportion of the SCC [31,32]. The target compressive strength of SCC was 69 MPa, which is standard for concrete of strength grade C60 with a guarantee rate of 95%. The target workability of the fresh SCC was a slump larger than 260 mm, a slump flow of 700 mm, and a flow time *T*_500_ less than 5 s. The target pouring quality of the fresh SCC was a density greater than 2450 kg/m^3^.

A total of 11 groups of mixtures for SCC were designed, with the influencing factors detailed in Table 5. The influencing factors included the ratio *R*_r_ of manufactured sand substituted by river sand, the ratios *R*_G_ and *R*_F_ of GGBS and fly ash in the total mass of the binders, and the maximum particle size of coarse aggregates (MPS). The water-to-binder ratio *w/b* was kept at 0.31, and the sand ratio *β*_s_ was kept constant at 50%.

The detailed mix proportions of the SCCs are presented in Table 6. The water content was 180 kg/m^3^. The water reducer was kept constant at 1.6% of the total mass of the binders. This is to eliminate the influence of the water reducer content on the fresh performance of the SCC.

## 3. Experimental Verification

### 3.1. Fresh Performance

**Figure 4:**
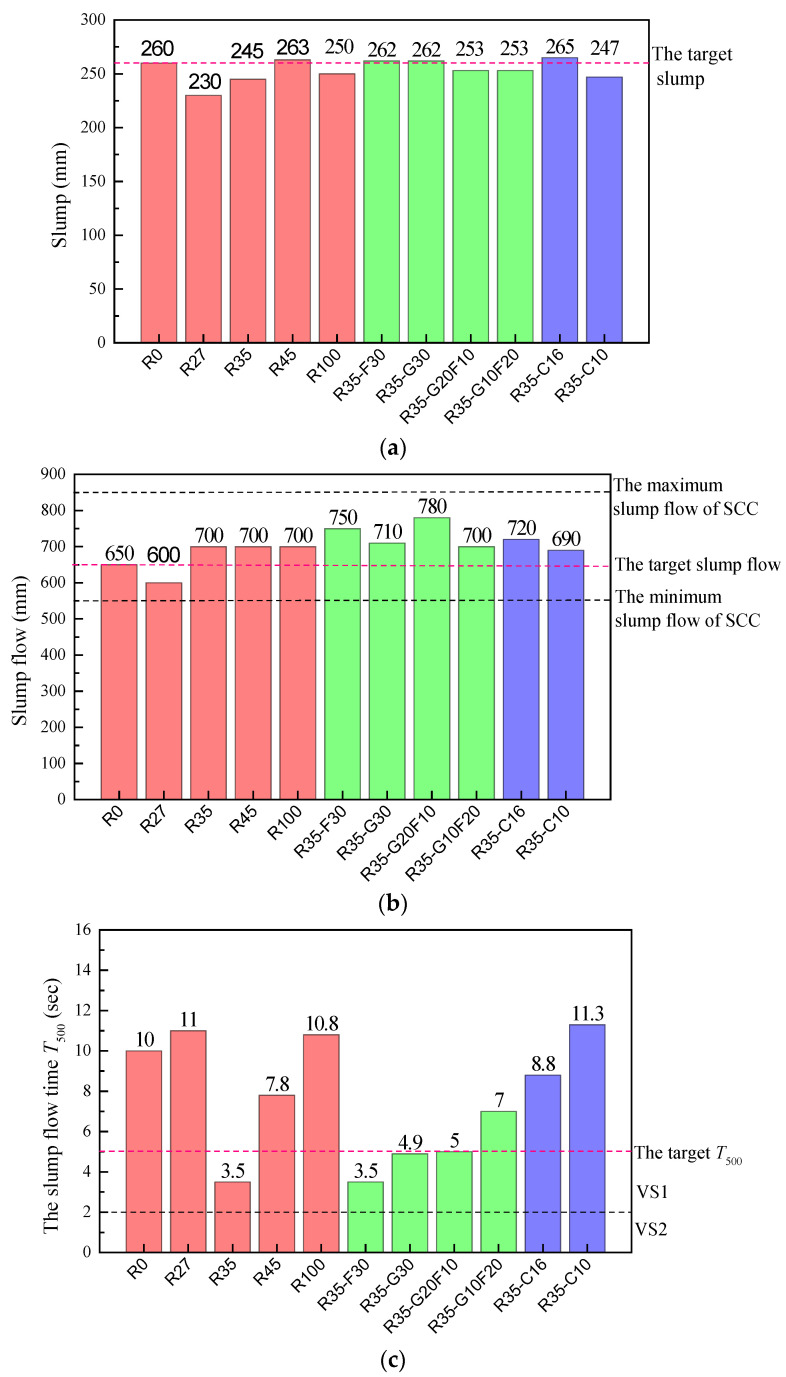
Workability of fresh SCC: (**a**) slump; (**b**) slump flow; (**c**) slump flow time *T*_500_.

A horizontal shaft forced mixer with a maximum capacity of 100 L was used to mix the SCC. The slump, slump flow and slump flow time *T*_500_ of the fresh SCC were tested immediately after mixing according to the Chinese code JGJ/T 283 [1], which is identical to ASTM C1611/C1611M [33]. The slump flow time *T*_500_ is the time from the lifting of the slump cone to the diameter of the slump expansion surface reaching 500 mm [34]. Results with comparisons to target indices and specified filling ability are presented in Figure 4.

The reasonable mixing of machine-made sand and river sand can optimize the particle grading of sand and bring about good workability for fresh SCC [14,15]. In this test, the workability of SCC first decreased with the increasing ratio of river sand up to 27.5%, then increased with the ratio of river sand from 27.5% to 45%. This is similar to the study of pumped concrete with manufactured sand and river sand [17]. Compared with R0, R45 and R100, R27 has the worst fresh performance, with a smaller slump of 230 mm, a lower slump flow of 600 mm and a longer flow time *T*_500_ of 11 s, while R35 has good fresh performance, with a suitable slump of 245 mm, a higher slump flow of 700 mm and a shorter flow time *T*_500_ of 3.5 s.

Fly ash and GGBS are conducive to the slump and slump flow of fresh SCC R35-F30 and R35-G30 compared to R35. However, the addition of GGBS prolongs the *T*_500_, while the addition of fly ash has no obvious influence on the *T*_500_. The *T*_500_ of R35-G30 is 1.43 times that of R35, while the *T*_500_ of R35-G30 was equal to that of R35. Compared with R35-G30, R35-F30 had the same slump, a higher slump flow and a shorter *T*_500_. This indicates that fly ash improved the working performance of the SCC. The reason is mainly due to the special morphological effects of fly ash, with microspheres featuring smooth surfaces, fine particle size and dense texture. Then, the fresh SCC with fly ash would adsorb a small amount of water in the mixing process [35], reducing the internal friction resistance and improving the flowability of the fresh concrete [8,36].

Compared with R35, R35-G10F20 and R35-G20F10 presented with a higher slump and slump flow. This indicated that the hybrid fly ash and GGBS contributed to the improvement of flowability of SCC, although no superposition effect of fly ash and GGBS was observed in these indexes. However, the negative superposition effect of hybrid fly ash and GGBS appeared on the *T*_500_. The *T*_500_ of R35-G10F20 and R35-G20F10 were longer than both R35-F30 and R35-G30.

Compared with R35, R35-C16 had a better flowability, with a higher slump of 265 mm, a higher slump flow of 720 mm and a flow time *T*_500_ of 8.8 s, while R35-C10 had a similar slump, a slightly lower slump flow and the longest *T*_500_ of 11.3 s. This indicates that the gradation of the coarse aggregates had effects on the slump and slump flow, and especially on the flow time of the fresh SCC. The *T*_500_ of the SCC increased with the decreasing maximum particle size of the coarse aggregate.

### 3.2. The Density

**Figure 5:**
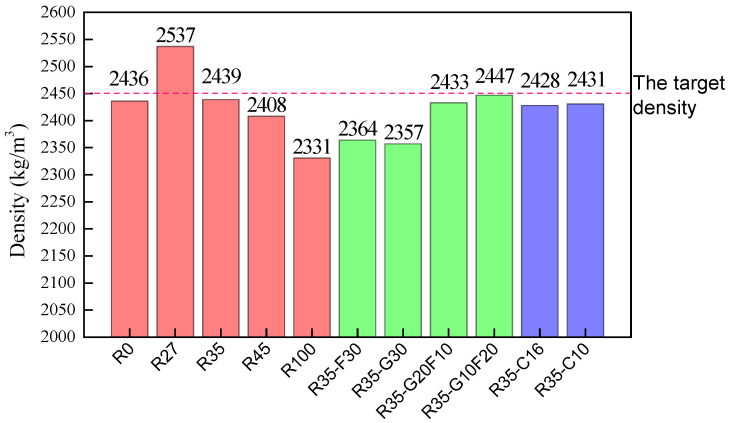
The densities of the SCCs.

The density is an important index to present the self-compacting quality, which affects the performance of hardened SCC. Cubic specimens with dimensions of 150 mm were weighed after being cast for 24 h. The densities of the SCCs were obtained from the weight divided by the volume of the cubic specimen. Results with a comparison of the target values are presented in Figure 5.

Comparing R0 to R100, the SCC with manufactured sand had a density that was 4.5% higher than the SCC with river sand. This came from the lower density of river sand presented in Table 3. The mixing of manufactured sand and river sand in appropriate proportions could increase the density of SCC. The density of SCC first increased with the increasing ratio of river sand up to 27.5%, then decreased with the ratio of river sand from 27.5% to 100%. This indicates a hybrid effect of finer river sand to improve the particle grading of manufactured sand to promote the density of SCC. Compared with R0, R45 and R100, R27 had a maximum density of 2537 kg/m^3^; R35 was next, with a density of 2439 kg/m^3^.

Compared to R35, R35-F30 and R35-G30 had a density decrease of about 3%. R35-G10F20 and R35-G20F10, R35-C10 and R35-C16 had an almost equal density as R35. This indicates a slight decrease in the density of SCC with the single addition of fly ash and GGBS. The hybrid addition of GGBS and fly ash and the MPS of the coarse aggregate had no influence on the density of SCC.

### 3.3. The Strength

**Figure 6:**
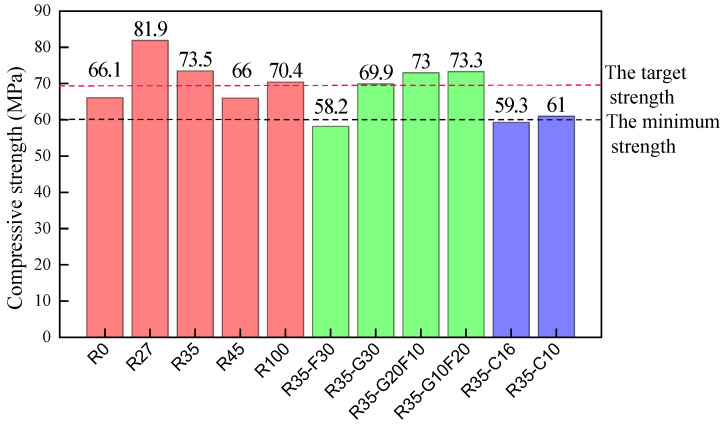
The cubic compressive strength of SCC.

The cubic compressive strengths of the SCCs at a curing age of 28 days were tested in accordance with China code GB/T50081 [37], which is identical to British Standard BS EN 12390-3 [38]. Cubic specimens with dimensions of 150 mm were used. Three specimens were tested as a group. Results are presented in Figure 6.

The substitution of manufactured sand with river sand could improve the cubic compressive strength of SCC. Except for R45, other SCCs with ratios of river sand of 27.5% and 35% had higher compressive strength; specifically, their compressive strength was increased by 23.9% and 11.2% compared to R0. Meanwhile, R100 had a higher cubic compressive strength, which was about 6.5% greater than R0. This indicates the good relationship of cubic compressive strength with the density of SCCs with hybrid sand. Manufactured sand is characterized by its rough surface, irregular particle shape, angular edges and the inevitable presence of stone powder, while river sand is characterized by smooth, round and small particles [39]. This different morphology between manufactured sand and river sand could influence the microstructure of SCCs to present their corresponding cubic compressive strengths.

The compressive strength of R35-F30 was only 79.0% that of R35. This is due to the significant dilution effect of fly ash with a lower strength activity index of 84.3% [40,41,42]. Comparatively, due to the strength activity index of GGBS, which reached 97.6%, the compressive strength of R35-G30 was reduced only by 4.9% compared to R35. Meanwhile, R35-G10F20 and R35-G20F10 had similar compressive strengths to R35. This indicates that the hybrid of fly ash and GGBS had a positive superposition effect on the compressive strength of SCC [11,43].

The maximum particle size of the coarse aggregate had a significant effect on the compressive strength of the SCC. Compared to R35, with a maximum particle size of 20 mm, the compressive strengths of R35-C16 and R35-C10, with maximum respective particle sizes of 16 mm and 10 mm, were reduced by 19.3% and 17.0%. This is similar to the compressive strength observed in conventional vibrated concrete [41]. Under the same mix proportion, the skeleton effect of coarse aggregates in concrete is weakened with decreasing MPS due to the lack of rationally closed packing among coarse aggregates with a similar particle size. At the same time, the decreased compressive strength was also related to the increased crushing index of smaller coarse aggregates, as presented in Table 4.

## 4. Optimization by Grey Relational Analysis

Grey relational analysis is a modern mathematics method for factor analysis in a system [26]. It is always used to evaluate the significance of factors in a given system and to judge the factors in order of importance. Its fundamental principle is evaluating the level of similarity for a data sequence by relevancy calculations. A greater relevancy means a higher similarity. In this paper, grey relational analysis is conducted based on the test results as the sample data to select the optimal mixtures from these eleven groups of SCC mixtures.

Assuming that the original data is *D* in the sequence of slump, slump flow, *T*_500_, density and cubic compressive strength, then,

(1) $$D=\left[\begin{array}{cccc} d_{1}^{1} & d_{2}^{1} & \ldots & d_{n}^{1}\\ d_{1}^{2} & d_{2}^{2} & \ldots & d_{n}^{2}\\ \vdots & \vdots & & \vdots \\ d_{1}^{m} & d_{2}^{m} & \ldots & d_{n}^{m} \end{array}\right]=\left[\begin{array}{c} \begin{array}{ccccc} 260 & 650 & 10.0 & 2436 & 66.1\\ 230 & 600 & 11.0 & 2537 & 81.9\\ 245 & 700 & 3.5 & 2439 & 73.5\\ 263 & 700 & 7.8 & 2408 & 66.0\\ 250 & 700 & 10.8 & 2331 & 70.4\\ 262 & 750 & 3.5 & 2364 & 58.2\\ 262 & 710 & 4.9 & 2357 & 69.9\\ 253 & 780 & 5.0 & 2433 & 73.0\\ 253 & 700 & 7.0 & 2447 & 73.3\\ 265 & 720 & 8.8 & 2428 & 59.3\\ 247 & 690 & 11.3 & 2431 & 61.0 \end{array} \end{array}\right]$$

where *d_k_^i^* is the original data of the index *k* at the solution *i*. *m* = 11 and *n* = 5 in this paper.

In this study, the slump, slump flow, and slump flow time *T*_500_ are the dominant indicators for the workability of fresh SCC. The slump and slump flow are larger for better responses, while the slump flow time *T*_500_ is smaller for a better response. The density and the compressive strength are the dominant responses for the hardened SCC, and they are larger for better responses. Thus, the standard matrix *C* can be obtained with the standardizing Equations (2)–(4),

(2) $$C_{k}^{i}=\frac{d_{k}^{i}-\min\left(d_{k}^{i},\;i=1,2,\ldots m\right)}{\max\left(d_{k}^{i},\;i=1,2,\ldots m\right)-\min\left(d_{k}^{i},\;i=1,2,\ldots m\right)}\;(\mathrm{for}\;\mathrm{the}\;\mathrm{larger}\;\mathrm{and}\;\mathrm{better}\;\mathrm{response})$$

(3) $$C_{k}^{i}=\frac{\max\left(d_{k}^{i},\;i=1,2,\ldots m\right)-d_{k}^{i}}{\max\left(d_{k}^{i},\;i=1,2,\ldots m\right)-\min\left(d_{k}^{i},\;i=1,2,\ldots m\right)}\;(\mathrm{for}\;\mathrm{the}\;\mathrm{smaller}\;\mathrm{and}\;\mathrm{better}\;\mathrm{response})$$

(4) $$C=\left[\begin{array}{cccc} C_{1}^{1} & C_{2}^{1} & \ldots & C_{5}^{1}\\ C_{1}^{2} & C_{2}^{2} & \ldots & C_{5}^{2}\\ \vdots & \vdots & & \vdots \\ C_{1}^{11} & C_{2}^{11} & \ldots & C_{5}^{11} \end{array}\right]=[\begin{array}{ccccc} 0.8571 & 0.2778 & 0.1667 & 0.5097 & 0.3333\\ 0.0000 & 0.0000 & 0.0385 & 1.0000 & 1.0000\\ 0.4286 & 0.5556 & 1.0000 & 0.5243 & 0.6456\\ 0.9429 & 0.5556 & 0.4487 & 0.3738 & 0.3291\\ 0.5714 & 0.5556 & 0.0641 & 0.0000 & 0.5148\\ 0.9143 & 0.8333 & 1.0000 & 0.1602 & 0.0000\\ 0.9143 & 0.6111 & 0.8205 & 0.1262 & 0.4937\\ 0.6571 & 1.0000 & 0.8077 & 0.4951 & 0.6245\\ 0.6571 & 0.5556 & 0.4943 & 0.5631 & 0.6371\\ 1.0000 & 0.6667 & 0.3205 & 0.4709 & 0.0464\\ 0.4857 & 0.5000 & 0.0000 & 0.4854 & 0.1181 \end{array}]$$

where C* is the referenced sequence.

C* = [*C*_1_*, *C*_2_*, …, *C*_5_*] = [1.0000 1.0000 1.0000 1.0000 1.0000] (5)

The matrix *C* is the compared sequence, and the correlation coefficient *ξ*_j_(i) is obtained from Equation (6)

(6) $$\xi_{j}\left(i\right)=\frac{\underset{j}{min}\underset{i}{min}\left|C_{i}^{*}-C_{i}^{j}\right|+\rho \underset{j}{max}\underset{i}{max}\left|C_{i}^{*}-C_{i}^{j}\right|}{\left|C_{i}^{*}-C_{i}^{j}\right|+\rho \underset{j}{max}\underset{i}{max}\left|C_{i}^{*}-C_{i}^{j}\right|}$$

where the value range of *ρ* is [0, 1], and generally takes the form of *ρ* = 0.5.

Then, the evaluation matrix of indexes *E* is shown as follows.

(7) $$E=\left[\begin{array}{cccc} \xi_{1}\left(1\right) & \xi_{1}\left(2\right) & \ldots & \xi_{1}\left(5\right)\\ \xi_{2}\left(1\right) & \xi_{2}\left(2\right) & \ldots & \xi_{2}\left(5\right)\\ \vdots & \vdots & & \vdots \\ \xi_{11}\left(1\right) & \xi_{11}\left(2\right) & \ldots & \xi_{11}\left(5\right) \end{array}\right]=\left[\begin{array}{ccccc} 0.7777 & 0.4091 & 0.3750 & 0.5049 & 0.4286\\ 0.3333 & 0.3333 & 0.3421 & 1.0000 & 1.0000\\ 0.4667 & 0.5294 & 1.0000 & 0.5125 & 0.5852\\ 0.8975 & 0.5294 & 0.4756 & 0.4440 & 0.4270\\ 0.5384 & 0.5294 & 0.3482 & 0.3333 & 0.5075\\ 0.8537 & 0.7500 & 1.0000 & 0.3732 & 0.3333\\ 0.8537 & 0.5625 & 0.7358 & 0.3640 & 0.4969\\ 0.5932 & 1.0000 & 0.7222 & 0.4976 & 0.5711\\ 0.5932 & 0.5294 & 0.4972 & 0.5337 & 0.5794\\ 1.0000 & 0.6000 & 0.4239 & 0.4859 & 0.3440\\ 0.4930 & 0.5000 & 0.3333 & 0.4928 & 0.3618 \end{array}\right]$$

We can then determine the entropy weight for the index *i* by assuming the number of indexes and evaluation objects are *n* = 5 and *m* = 11, respectively:

(8) $$f_{ij}=\frac{\xi_{j}\left(i\right)}{\sum_{j=1}^{m}\xi_{j}\left(i\right)}$$

Thus,

(9) $$F=\left[\begin{array}{cccc} f_{11} & f_{12} & \ldots & f_{1n}\\ f_{21} & f_{22} & \ldots & f_{2n}\\ \vdots & \vdots & & \vdots \\ f_{m1} & f_{m2} & \ldots & f_{mn} \end{array}\right]=\left[\begin{array}{ccccc} 0.105 & 0.065 & 0.060 & 0.091 & 0.076\\ 0.045 & 0.053 & 0.055 & 0.180 & 0.177\\ 0.063 & 0.084 & 0.160 & 0.092 & 0.104\\ 0.121 & 0.084 & 0.076 & 0.080 & 0.076\\ 0.073 & 0.084 & 0.056 & 0.060 & 0.090\\ 0.115 & 0.120 & 0.160 & 0.067 & 0.059\\ 0.115 & 0.090 & 0.118 & 0.066 & 0.088\\ 0.080 & 0.159 & 0.115 & 0.090 & 0.101\\ 0.080 & 0.084 & 0.080 & 0.096 & 0.103\\ 0.135 & 0.096 & 0.068 & 0.088 & 0.061\\ 0.067 & 0.080 & 0.053 & 0.089 & 0.064 \end{array}\right]$$

The entropy *h*_i_ of the index *i* is shown as

(10) $$h_{i}=-k\overset{11}{\underset{j=1}{\sum }}f_{ij}lnf_{ij}$$

where *k* = 1/*ln*m = 1/*ln*11 = 0.417.

Thus,

H = [*h*_1_, *h*_2_, …, *h*_5_] = [0.98044, 0.98323, 0.96412, 0.97988, 0.97778](11)

The entropy weight for the index *i* is

(12) $$\omega_{i}=\frac{1-h_{i}}{n-\sum_{1}^{n}h_{i}}\left(0\leq \omega_{i}\leq 1,\sum_{i=1}^{n}\omega_{i}=1\right)$$

The entropy weight matrix of various indexes is

W = [*ω*_1_, *ω*_2_, …, *ω*_5_] = [0.17076, 0.14638, 0.31319, 0.17567, 0.19400](13)

Therefore, the comprehensive evaluation index is

*R* = *E* × *W^T^*
= [0.48197, 0.58253, 0.67393, 0.54055, 0.43551, 0.69897, 0.61890, 0.67207, 0.54066, 0.54344, 0.41853](14)

This means the sequence is R35-F30, R35, R35-G20F10, R35-G30, R27, R35-C16, R35-G10F20, R45, R0, R100, and R35-C10. The mixtures of R35-F30, R35 and R35-G20F10 are the relatively better groups of the eleven mixtures.

## 5. Discussion

Based on the test results of eleven SCCs in this study, their workability meets the requirements to be labeled as self-compacting. The SCCs marked as R27, R35, R100, R35-G30, R35-G20F10 and R35-G10F20 meet the target compressive strength of the SCC. Due to the complex influences of the properties and contents of raw materials, including fly ash, GGBS, manufactured sand, river sand and coarse aggregate, different optimal mixtures can be obtained by considering the performance. Considering the slump and the slump flow of fresh SCC, the mixtures R0, R45, R35-F30, R35-G30 and R35-C16 are optimal. Considering the flow time *T*_500_ of fresh SCC, the mixtures R35, R35-F30 and R35-G20F10 are optimal. Considering the density of SCC, the mixtures R0, R27, R35 and R35-G10F20 are optimal. Considering the compressive strength of SCC, the mixtures R27, R35, R100, R35-G30, R35-G20F10 and R35-G10F20 are optimal.

Therefore, it is difficult to determine the optimal mixture of SCC only by the test results to satisfy all aspects of performance. In this condition, grey relational analysis is used to obtain the optimal solution among these eleven SCC mixtures after comprehensively considering the workability, density and mechanical properties of the SCCs. As a result, R35-F30, R35 and R35-G20F10 are the better of the eleven mixtures. This agrees well with the experimental results. R35-F30 has a fresh performance with a high slump of 262 mm, a high slump flow of 750 mm and a short flow time *T*_500_ of 3.5 s, a density of 2364 kg/m^3^, and a compressive strength of 58.2 MPa. R35 has a fresh performance with a suitable slump of 245 mm, a high slump flow of 700 mm and a short flow time *T*_500_ of 3.5 s, a density of 2439 kg/m^3^, and a high compressive strength of 73.5 MPa. R35-G20F10 has a fresh performance with a high slump of 253 mm, a high slump flow of 780 mm, a short flow time T500 of 5 s, and a density of 2433 kg/m3 and a high compressive strength of 73.0 MPa.

Given all of the above, the experimental study is suitable for comparing the effects of the raw materials on one aspect of the performance of SCCs, such as workability, density or compressive strength. The grey relational analysis used in this study is applicable to further optimize the mix proportion of SCCs while considering the comprehensive performance of the SCC.

## 6. Conclusions

Based on the absolute volume method of mix proportion design of SCC, eleven groups of SCCs were designed with changes in the content of fly ash and GGBS, the content of manufactured sand and river sand, and the maximum particle sizes of coarse aggregates. The design was verified by experimental studies and evaluated by grey relational analysis. Conclusions can be drawn as follows.

The replacement of manufactured sand by a certain amount of river sand could improve the flowability of fresh SCC and increase the compressive strength of hardened SCC. Both the GGBS and fly ash are beneficial for the slump and slump flow of fresh SCC; however, they also increase the flow time. The hybrid effect of GGBS with fly ash would improve the workability of SCC with equal compressive strength. The maximum particle size of the coarse aggregate significantly influenced the performance of the SCC. Compared to an SCC with a maximum coarse aggregate particle size of 20 mm, an SCC with a maximum coarse aggregate particle size of 16mm demonstrated more favorable workability, but the cubic compressive strength of SCC decreased by nearly 20%.

Based on the test results, a grey correlation analysis model was built. Combined with the entropy evaluation method, the optimal mixtures of SCC were selected from these eleven group mixtures. The optimal mixture considering the workability of fresh SCC was found to be R35-F30, which demonstrated fresh performance with a slump of 265 mm, a slump flow of 750 mm and a flow time *T*_500_ of 3.5 s. The optimal mixture when comprehensively considering the workability and mechanical properties of SCC was found to be R35, which demonstrated fresh performance with a slump of 245 mm, a slump flow of 700 mm, a flow time *T*_500_ of 3.5 s, a density of 2439 kg/m^3^ and a compressive strength of 73.5 MPa. This method has improved the objectivity of the evaluation and avoided the loss of information which can be used for the optimization of mix proportions of SCC.

## Figures and Tables

**Table 1:** Physical and mechanical properties of cement.

Fineness (45 μm, %)	Water Requirement of Normal Consistency (%)	Density (kg/m^3^)	Setting Time (min)		Compressive Strength (MPa)		Flexural Strength (MPa)	
			Initial	Final	3 Days	28 Days	3 Days	28 Days
8.6	26.6	3195	176	222	34.9	57.8	6.23	8.83

**Table 2:** Physical and mechanical properties of fly ash and GGBS.

No.	Fineness (45 μm, %)	Density (kg/m^3^)	Water Demand Ratio (%)	Specific Surface (cm^2^/g)	Strength Activity Index (%)	Loss on Ignition (%)
Fly ash	6.9	2280	95	3590	84.3	5.9
GGBS	1.0	2998	-	4388	97.6	2.9

**Table 3:** Physical and mechanical properties of fine aggregates.

Type	Fineness Modulus	Apparent Density (kg/m^3^)	Closed Packing Density (kg/m^3^)	Bulk Density (kg/m^3^)	Stone Powder Content (%)	Water Absorption (%)
Manufactured sand	2.78	2730	1698	1923	7.45	0.25
River sand	2.00	2597	1477	1641	-	0.15

**Table 4:** Physical and mechanical properties of coarse aggregates.

Maximum Particle Size (mm)	Apparent Density (kg/m^3^)	Pile-Up Density (kg/m^3^)		Crushed Index (%)	Content of Needle-Pieces (%)
		Loose	Close		
20	2770	1583	1682	7.2	4.70
16	2766	1575	1662	9.6	4.54
10	2711	1513	1575	16.8	1.32

**Table 5:** The influencing factors designed in the mix proportion of the SCCs.

No	*w*/*b*	*β*_s_ (%)	*R*_G_ (%)	*R*_F_ (%)	*R*_r_ (%)	MPS (mm)
R0	0.31	50	0	0	0	20
R27					27.5	
R35					35	
R45					45	
R100					100	
R35-F30	0.31	50	0	30	35	20
R35-G30			30	0		
R35-G20F10			20	10		
R35-G10F20			10	20		
R35-C16	0.31	50	0	0	35	16
R35-C10						10

**Table 6:** The detailed mix proportions of the SCCs (kg/m^3^).

No	Water	Cement	Mineral Admixture		Coarse Aggregate			Fine Aggregate		Water Reducer
			GGBS	Fly Ash	16~20	10~16	5~10	Manufactured Sand	River Sand	
R0	180	581	0	0	247	206	370	822	0	9.29
R27	180	581	0	0	247	206	370	596	226	9.29
R35	180	581	0	0	247	206	370	534	288	9.29
R45	180	581	0	0	247	206	370	452	370	9.29
R100	180	581	0	0	247	206	370	0	822	9.29
R35-F30	180	406	0	174	247	206	370	534	288	9.29
R35-G30	180	406	174	0	247	206	370	534	288	9.29
R35-G20F10	180	406	116	58	247	206	370	534	288	9.29
R35-G10F20	180	406	58	116	247	206	370	534	288	9.29
R35-C16	180	581	0	0	0	294	528	534	288	9.29
R35-C10	180	581	0	0	0	0	822	534	288	9.29

## Data Availability

The data presented in this study are available in the submitted article.
